# Bibliometric Analysis of the Post‐Stroke Depression (1985–2024)

**DOI:** 10.1002/brb3.71345

**Published:** 2026-04-27

**Authors:** Xinjuan Wang, Yinfen Qian, Qi He

**Affiliations:** ^1^ Department of Nursing School of Medicine Taizhou University Taizhou China; ^2^ Department of Cardiovascular Medicine Taizhou Central Hospital (Taizhou University Hospital) Taizhou China; ^3^ School of Nursing Xiangnan University Chenzhou China

**Keywords:** bibliometric analysis, mental health, post‐stroke depression, stroke rehabilitation

## Abstract

**Introduction:**

This bibliometric analysis examines the evolution of research into post‐stroke depression (PSD) between 1985 and 2024, evaluating publication trends, key contributions, and thematic shifts in the field.

**Methods:**

The study analyzed 3146 studies retrieved from the Web of Science Core Collection. Keyword and thematic analyses were conducted using the software tools VOSviewer and CiteSpace.

**Results:**

Publications on PSD increased significantly after 2010. China (796 articles) and the United States (615 articles) were the leading countries in research output. Analyses revealed core research themes encompassing depression prevalence, rehabilitation strategies, quality of life, and recovery outcomes. Thematic clusters identified diverse areas of focus, including cognitive impairment, socioeconomic disparities, caregiver roles, alternative therapies, and the influence of culture on PSD management. Emerging trends indicated by citation bursts included “healthcare professionals,” “fatigue,” and “meta‐analysis,” pointing to current frontiers in interdisciplinary care, symptom complexity, and evidence synthesis. Highly cited studies emphasized integrating mental health assessments into stroke care and the critical role of rehabilitation in mitigating PSD.

**Conclusion:**

The findings illustrate the field's progression from early observational studies to multifaceted investigations addressing the biological, psychological, and social dimensions of PSD. Future research should prioritize multidisciplinary frameworks, culturally sensitive interventions, and innovative therapies. This study underscores the need for holistic rehabilitation models that integrate physical and mental healthcare to improve outcomes for stroke survivors, thereby informing clinical practice, policy, and further research.

## Graphical abstract

This bibliometric analysis maps the evolution of post‐stroke depression (PSD) research from 1985 to 2024. By analyzing 3146 publications from the Web of Science Core Collection using VOSviewer and CiteSpace, the study delineates global publication trends, collaborative networks, and the dynamic shifts in research themes over four decades. Research output surged after 2010, with China and the United States being the leading contributors. The analysis reveals a progression from initial focus on prevalence and risk factors to contemporary investigations into rehabilitation, quality of life, and comorbid conditions like cognitive impairment. The field has matured from descriptive observation to a multifaceted, biopsychosocial understanding of PSD. The findings underscore the necessity for holistic rehabilitation models that integrate mental and physical health care. Future research should prioritize culturally sensitive interventions and innovative therapies to improve long‐term outcomes for stroke survivors.

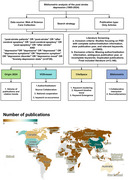

## 1 Introduction

Stroke is the second leading cause of death and the leading cause of adult disability worldwide, affecting over 13 million people every year (Kapoor, Si, et al. 2019; Xu and Liang 2021; Dajpratham et al. 2020). The residual neurological dysfunction and socio‐economic burden it causes have become major public health challenges in the 21st century (Williams et al. 2020; Wijeratne and Sales 2021; Werheid 2016). According to World Health Organization data, a stroke occurs every 4 s globally, with approximately 75% of survivors experiencing varying degrees of cognitive impairment, motor dysfunction, or psychological issues (Douven et al. 2017; Baker et al. 2018; Bucur and Papagno 2018). PSD is a neuropsychiatric complication that has attracted increasing attention from the academic community. This is due to the high incidence rate of the condition and the serious harm it inflicts on patients (Y. Shi et al. 2017; Chen et al. 2020; Zhang et al. 2017). A substantial body of research has demonstrated that between 30% and 50% of stroke patients will exhibit depressive symptoms within one year of the onset of their stroke. Furthermore, severe depressive disorders have been found to account for 15%–25% of cases (Kim et al. 2015; Ling et al. 2020; Baker et al. 2021). This depressive state, which is secondary to brain injury, has been shown to have a number of consequences. First, it has been demonstrated to significantly reduce patients' rehabilitation compliance. Second, it may exacerbate neurological deficits through neuroendocrine disorders, thus forming a vicious cycle of “depression dysfunction.” Finally, this may ultimately lead to a 3–4 times higher mortality rate in patients compared to non‐depressed stroke patients (Z. M. Shi et al. 2023; Zhao et al. 2019; Mayman et al. 2021).

Following the seminal study in 1985, which demonstrated the biological correlation between stroke and depression, research into PSD has evolved from simple observations of symptoms to a multidisciplinary exploration of the underlying mechanisms (Dong et al. 2020; H. P. Shen et al. 2019; Li et al. 2017). The advent of neuroimaging technology has facilitated researchers in accurately localizing the correlation between structural alterations in pivotal brain regions, such as the prefrontal cortex and the limbic system, and depressive symptoms. Advancements in molecular biology have elucidated the pivotal function of molecular pathways, including the imbalance of the glutamatergic system and the surge of neuroinflammatory factors, in the genesis of PSD (Kapoor, Lanctot, et al. 2019; J. J. Wang et al. 2021; Song et al. 2015). At the clinical practice level, the American Heart Association (AHA) released the “Stroke Rehabilitation Guidelines” in 2020. This was a significant development in the field, as it was the first time that depression screening was listed as a mandatory assessment item. This marked a shift in the management of PSD from adjuvant therapy to systematic intervention (Zeng et al. 2017; S. S. Wang et al. 2016; Shao et al. 2015). However, significant knowledge gaps persist in extant research. First, diagnostic criteria have not been unified (see DSM‐5 and ICD‐11 for classification differences). Second, a long‐standing debate between the “vascular hypothesis” and the “psychological stress hypothesis” regarding pathological mechanisms persists. Third, controversies surround drug treatment plans regarding the efficacy of selective serotonin reuptake inhibitors (SSRIs) (Wei et al. 2016; X. Y. Shen et al. 2017; Hordacre et al. 2021; Cheng et al. 2021). These contradictions underscore the pressing necessity for a systematic review of the evolutionary trajectory of knowledge in this field.

The present study is grounded in the theoretical framework of scientometrics. It conducts a panoramic analysis of PSD research literature from 1985 to 2024, intending to achieve three core objectives. First, it seeks to depict the global research pattern and knowledge diffusion characteristics of this field through literature output trends and geographic distribution maps. Second, it uses co‐word analysis and clustering algorithms to identify the historical evolution of research hotspots and the trends of interdisciplinary integration. Finally, it constructs an author collaboration network and institutional influence model to analyze the distribution patterns of scientific research productivity and knowledge innovation mechanisms. To achieve these objectives, the Web of Science Core Collection (WOSCC) was selected as the exclusive data source—an internationally recognized database for bibliometric research, ensuring comprehensive coverage of high‐impact, peer‐reviewed PSD literature. Tools, including VOSviewer and CiteSpace, were utilized for multi‐level bibliometric analysis, and Altmetric indicators were introduced to evaluate the social dissemination effect of research results.

It is important to note that this study is the first to extend the time span of bibliometric analysis from 1985 to 2024, thereby encompassing the most recent advancements in cutting‐edge areas such as artificial intelligence‐assisted diagnosis and microbiota‐gut brain axis regulation. This comprehensive approach involves the incorporation of pre‐published literature and conference abstracts, thus providing a more extensive and up‐to‐date overview of the subject. This forward‐looking perspective not only compensates for the lagging shortcomings of traditional reviews but also provides data support for predicting the future direction of PSD research. The research results are disseminated to clinical workers and policymakers through a visual interactive platform, thereby effectively promoting knowledge transfer and evidence‐based practice.

## 2 Methods

### 2.1 Data Collection and Search Strategy

**FIGURE 1:**
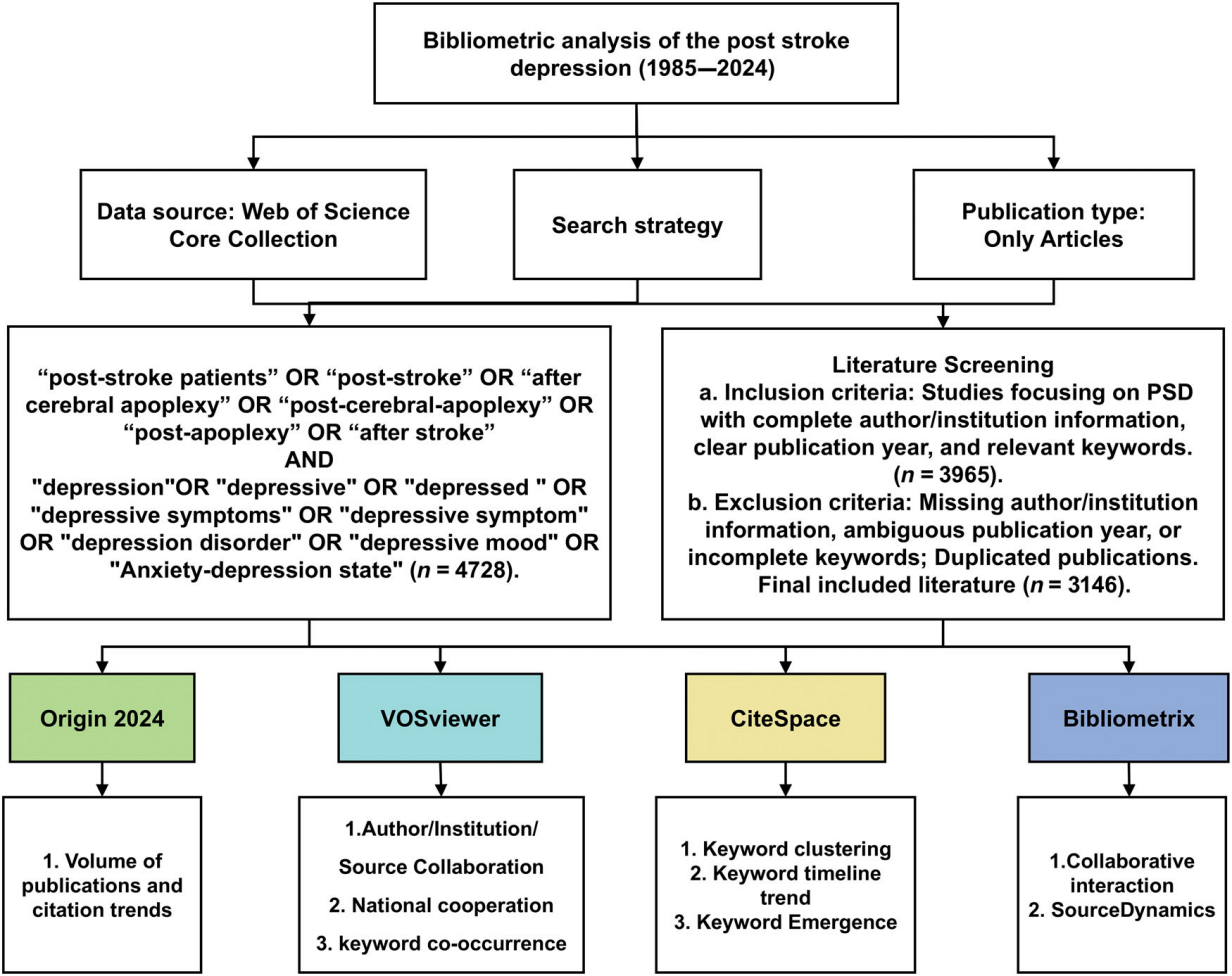
Search strategy and analysis content.

The Web of Science database is a comprehensive information service platform that facilitates interdisciplinary literature searches. The database is universally recognized for providing a large number of indexed publications that are often used for bibliometric analyses. In this study, the WOSCC was selected as the primary source of the literature. The time period searched was from 1985 to 2024. In order to guarantee the precision of the literature and subjects incorporated within the analysis, a title search was employed utilizing the following strategy: TS  = (“post‐stroke patients” OR “post‐stroke” OR “after cerebral apoplexy” OR “post‐cerebral‐apoplexy” OR “post‐apoplexy” OR “after stroke”) AND TS = (“depression” OR “depressive” OR “depressed ” OR “depressive symptoms” OR “depressive symptom” OR “depression disorder” OR “depressive mood” OR “Anxiety‐depression state”). The search strategy and analysis content are illustrated in Figure 1. A total of 3485 studies were retrieved, and then the literature titles were manually screened according to the established inclusion and exclusion criteria. These included incomplete information about authors and institutions, an ambiguous year of publication, as well as incomplete keywords and duplicated publications. Literature unrelated to the topic was also excluded, such as conference papers, news reports, and notification newsletters. A total of 3146 articles were found to export records and cite references in plain text file format. All data utilized in this study were obtained from public databases and thus did not necessitate ethics committee approval or informed consent.

### 2.2 Statistical Analysis

Annual publication charts were generated using Origin Pro 2025. For bibliometric visualization and analysis, VOSviewer 1.6.20 and CiteSpace 6.2R6 were employed. Core parameters influencing results are specified below:

*VOSviewer 1.6.20*: English literature data from WOS were imported; Overlay Visualization and Network Visualization were adopted. Key parameters: Association Strength was selected for normalization; clustering relied on the default VOS algorithm (modularity optimization) to ensure thematic accuracy.

*CiteSpace 6.2R6*: WOS data were exported as “Full Record with Cited References” (plain text format). Key parameters: 1‐year time slices (1985–2024); the cosine algorithm was used for network strength calculation; Top 10% of targets extracted per slice; keyword threshold (Top N) = 50, node extraction threshold (k‐metric) = 25; Pathfinder and pruned slice networks were used to optimize core network structures; the latent semantic indexing (LSI) algorithm was applied to generate cluster labels for keywords.

## 3 Results

### 3.1 Annual Publication Outputs and Trends

**FIGURE 2:**
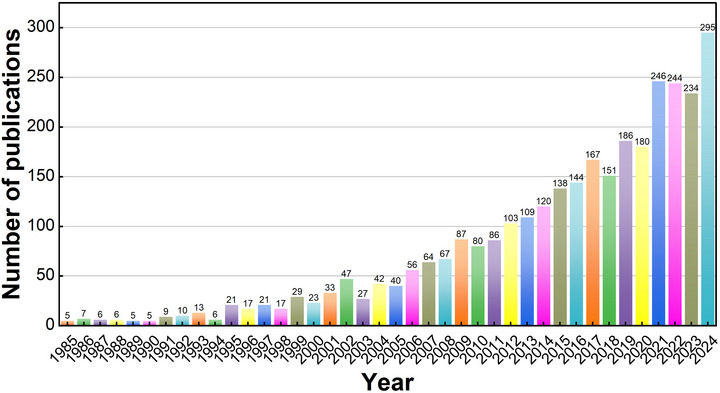
Annual number of publications and trend.

As shown in Figure 2, the annual publication trend of PSD research (1985–2024) exhibits three distinct phases with clear inflection points. The first phase (1985–1994) was a foundational period with modest growth (5–10 publications/year), reflecting initial recognition of PSD as a clinical phenomenon. A critical inflection point emerged in 1995 (publications = 21, 217% increase from 1994), coinciding with landmark epidemiological studies that established PSD's high prevalence and long‐term impact on stroke recovery, driving broader clinical attention.

The second phase (1995–2009) saw steady growth, peaking at 47 publications in 2002, followed by moderate fluctuations. The third and most impactful inflection point occurred post‐2010, with a sharp acceleration (80 publications in 2010 to 186 in 2019) driven by three key factors: (1) the release of evidence‐based clinical guidelines (e.g., AHA 2020 guidelines) integrating PSD screening into routine stroke care; (2) methodological advances in neuroimaging and non‐pharmacological interventions (e.g., transcranial stimulation) enabling mechanistic and translational research; and (3) increased global funding for mental health integration in chronic disease management.

The trend reached a peak in 2021 (246 publications), likely amplified by heightened focus on post‐stroke mental health during the COVID‐19 pandemic, as isolation exacerbated depressive symptoms in survivors. A slight dip in 2022–2023 (244–234 publications) was followed by a robust rebound to 295 publications in 2024, underscoring the field's sustained relevance. This trajectory reflects the maturation of PSD research from descriptive epidemiology to targeted, guideline‐driven interventions, with ongoing growth aligned with unmet clinical needs for holistic stroke rehabilitation.

### 3.2 Detailed Analysis of Key Countries

**FIGURE 3:**
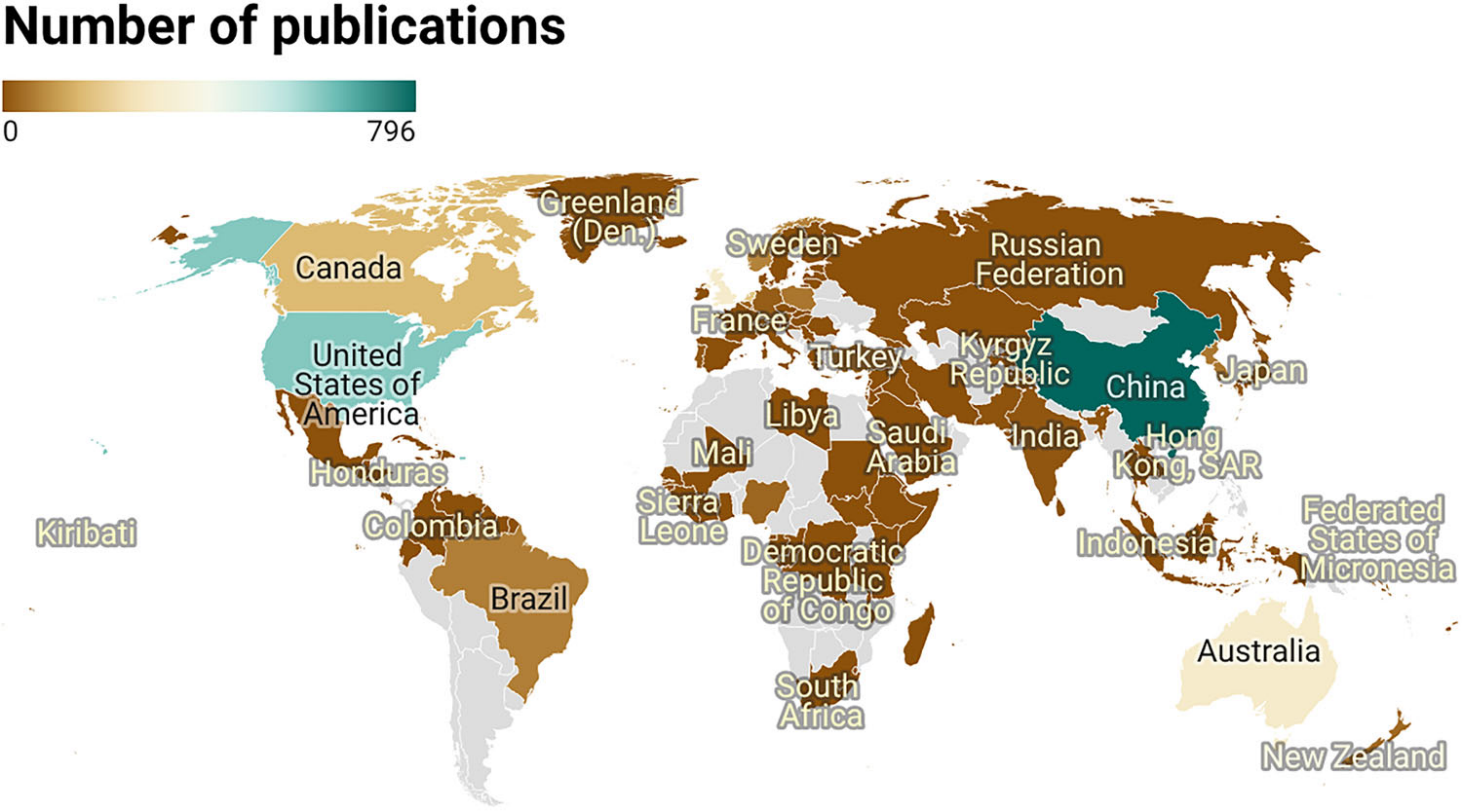
World map of the range of the main research countries.

**FIGURE 4:**
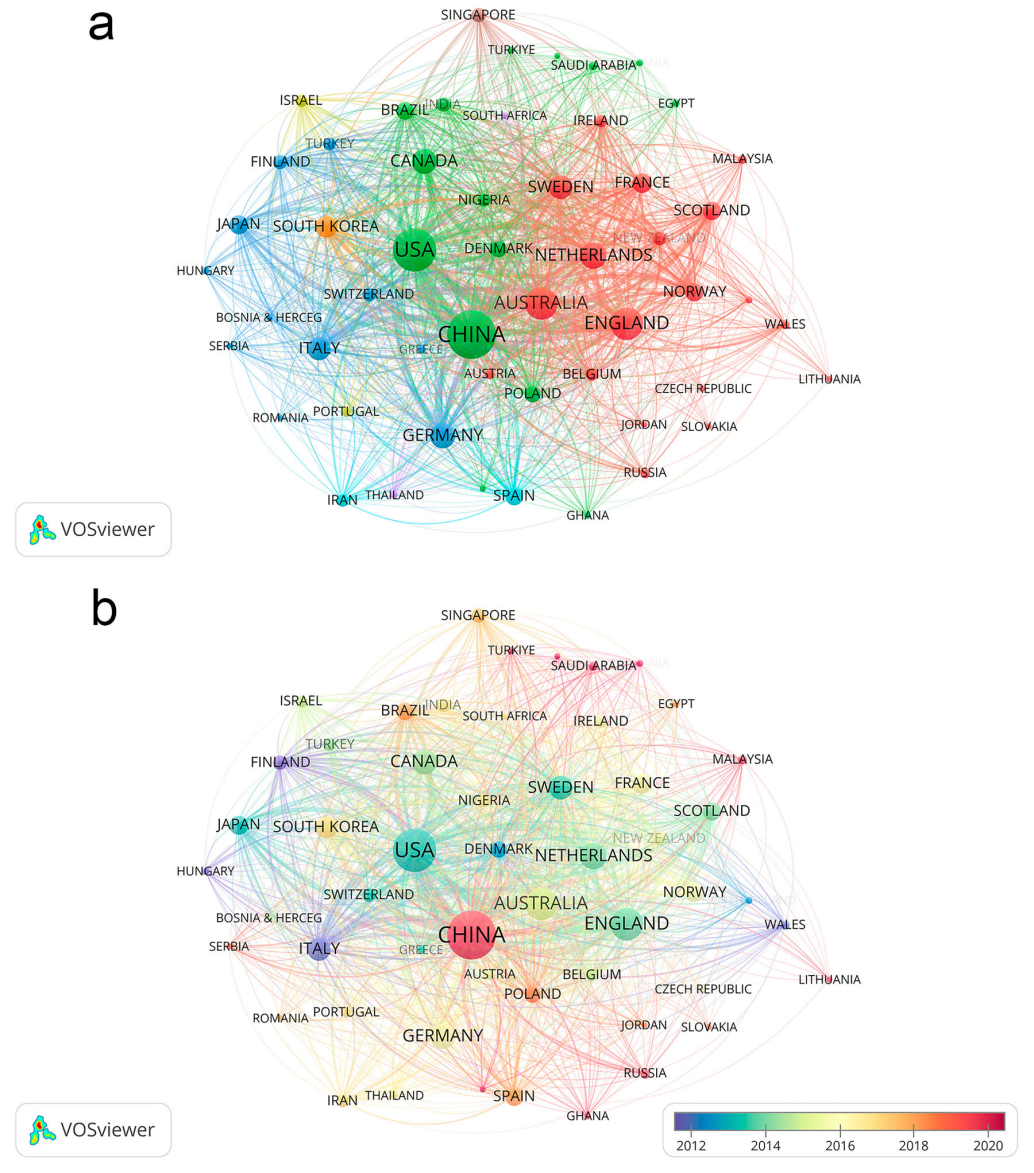
Country cooperation co‐occurrence map, (a) country cooperation co‐occurrence clustering map, (b) country cooperation co‐occurrence time view.

Figure 3, the global geographic distribution map, Figure 4a, the country cooperation co‐occurrence clustering map, Figure 4b, the country cooperation co‐occurrence temporal view, and Table 1 collectively reveal distinct global contribution patterns, hierarchical collaborative networks, and dynamic temporal evolutionary trends of post‐stroke depression (PSD) research over the 40‐year study period.

**TABLE 1:** Top 15 high‐output countries/regions.

Ranking	Country	Documents	Citations	Total link strength
1	China	796	11,542	467,805
2	USA	615	21,974	494,067
3	England	299	14,331	293,138
4	Australia	290	10,518	321,107
5	The Netherlands	178	7089	207,974
6	Germany	154	5415	154,866
7	Canada	153	7515	124,915
8	Sweden	139	5998	132,302
9	Italy	127	4455	127,088
10	South Korea	110	3104	108,093
11	France	83	3229	74,148
12	Japan	80	2264	70,013
13	Scotland	74	3517	82,515
14	Norway	71	2367	107,401
15	Brazil	64	1540	60,771

Figure 4a employs VOSviewer‐based hotspot clustering to visualize the thematic clustering structure and collaborative interconnections among countries. Two core clusters dominate the global research landscape. The first cluster centers on China with 796 publications, as detailed in Table 1, featuring dense internal collaborative links that demonstrate strong cohesion within its research network. The second cluster is led by the United States with 615 publications, as documented in Table 1, exhibiting extensive cross‐cluster collaborative connections with England, Australia, and the Netherlands. Figure 3, the global geographic distribution map, confirms the worldwide reach of PSD research, with key contributing countries distributed across Asia, North America, Europe, and Oceania. This geographic spread aligns closely with the clustering results presented in Figure 4a, contextualizing the spatial distribution of collaborative networks.

### 3.3 Analysis of Key Institutions

**FIGURE 5:**
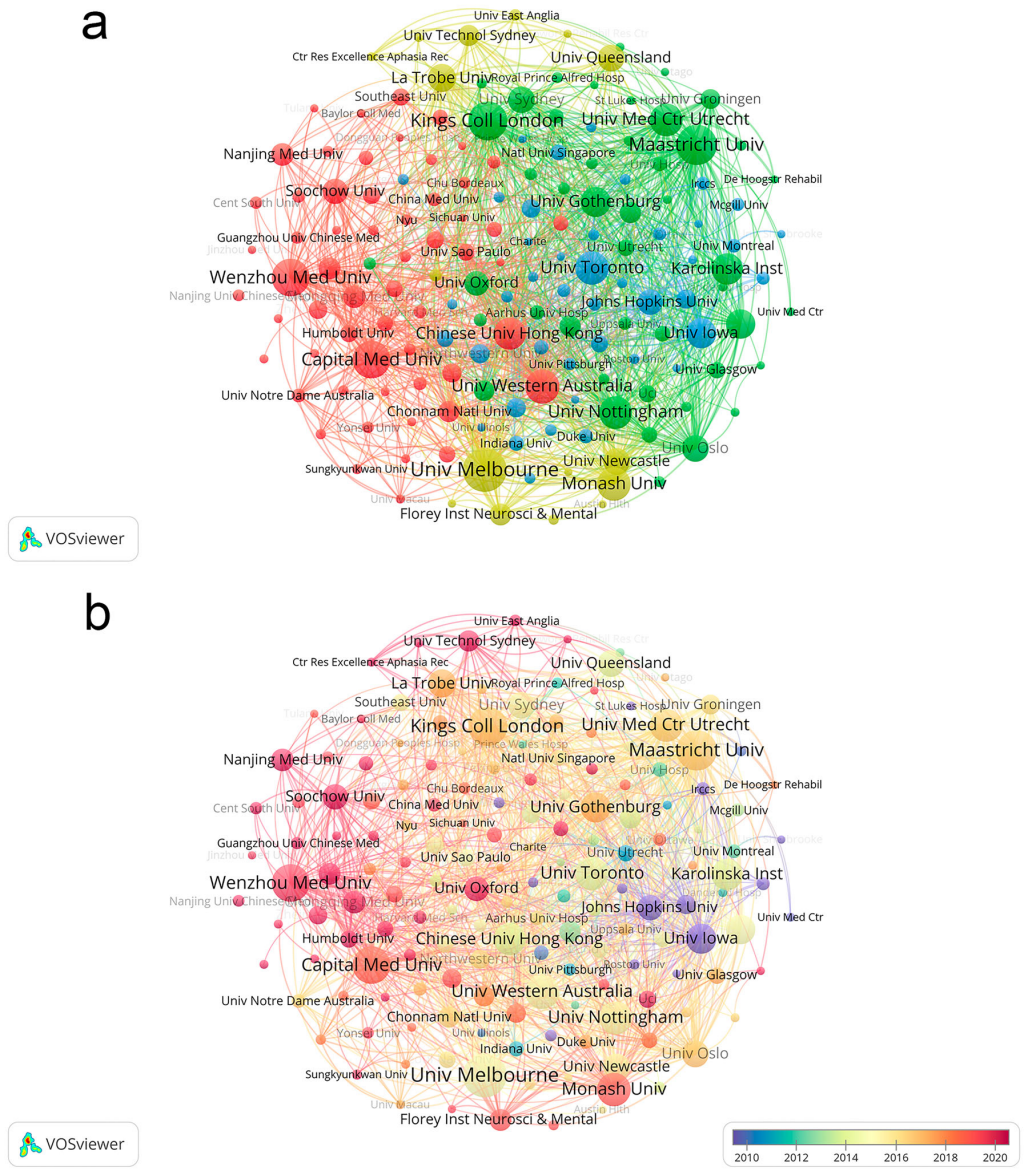
(a) Clustered co‐occurrence, (b) temporal co‐occurrence of research organization collaborations.

Figure 5a, the institutional collaboration co‐occurrence clustering map, Figure 5b, the institutional collaboration temporal view, and Table 2 collectively illustrate the static collaborative structures, core institutional contributions, and dynamic temporal evolution of PSD research across the 40‐year study period. These visualizations and data work synergistically to reveal the hierarchical network of research institutions and their evolving roles in advancing the field.

**TABLE 2:** Top 15 high‐output institutions.

Ranking	Organization	Documents	Citations
1	University of Melbourne	60	2195
2	Maastricht University	55	1379
3	King's College London	53	2487
4	Capital Medical University	51	965
5	Wenzhou Medical University	51	858
6	University of Western Australia	46	1777
7	Monash University	45	805
8	University of Toronto	45	1951
9	University Medical Center Utrecht	44	1930
10	University of Nottingham	44	1347
11	The Chinese University of Hong Kong	43	1232
12	Karolinska Institute	42	1322
13	University of Gothenburg	40	942
14	University of Iowa	40	3286
15	University of Edinburgh	39	2051

Figure 5a employs VOSviewer‐based hotspot clustering to visualize the thematic clustering structure and collaborative interconnections among research institutions. Three distinct core clusters characterize the global institutional collaboration landscape. The first cluster centers on the University of Melbourne, with 60 publications as detailed in Table 2, serving as a pivotal node with dense internal collaborative links that reflect strong cohesion within its associated research network. The second cluster includes Maastricht University and King's College London, with 55 and 53 publications, respectively, as documented in Table 2, and exhibits extensive cross‐institutional collaborative connections that span multiple research contexts. The third cluster features Capital Medical University and Wenzhou Medical University, each contributing 51 publications as indicated in Table 2, with prominent internal collaboration intensity that underscores the cohesive nature of their research networks. The University of Western Australia, Monash University, and the University of Toronto form secondary nodes within these core clusters, enhancing the overall connectivity of the global institutional collaboration network.

Figure 5b, the institutional collaboration temporal view, complements Figure 5a, by presenting the temporal distribution of institutional research activities through color gradients. The University of Melbourne maintains consistent high research activity across the entire 1985–2024 period, demonstrating a long‐standing and sustained contribution to the field. Maastricht University and King's College London exhibit stable research engagement from the early 2000s onwards, with their temporal color gradients reflecting continuous output over decades. Capital Medical University and Wenzhou Medical University show a notable increase in research activity from 2015 onwards, with a distinct deepening of color in the temporal view that corresponds to the surge in their publication output during this period, as detailed in Table 2. The University of Western Australia, Monash University, and the University of Toronto have displayed steady research activity since the mid‐2000s, forming reliable collaborative partners within the broader institutional network.

### 3.4 Journal Analysis

**FIGURE 6:**
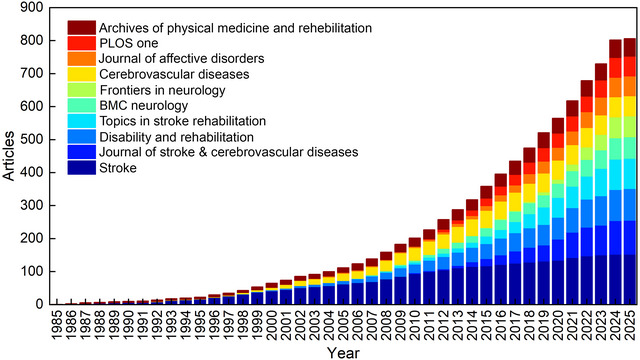
Journal publication volume and double figure overlay.

**FIGURE 7:**
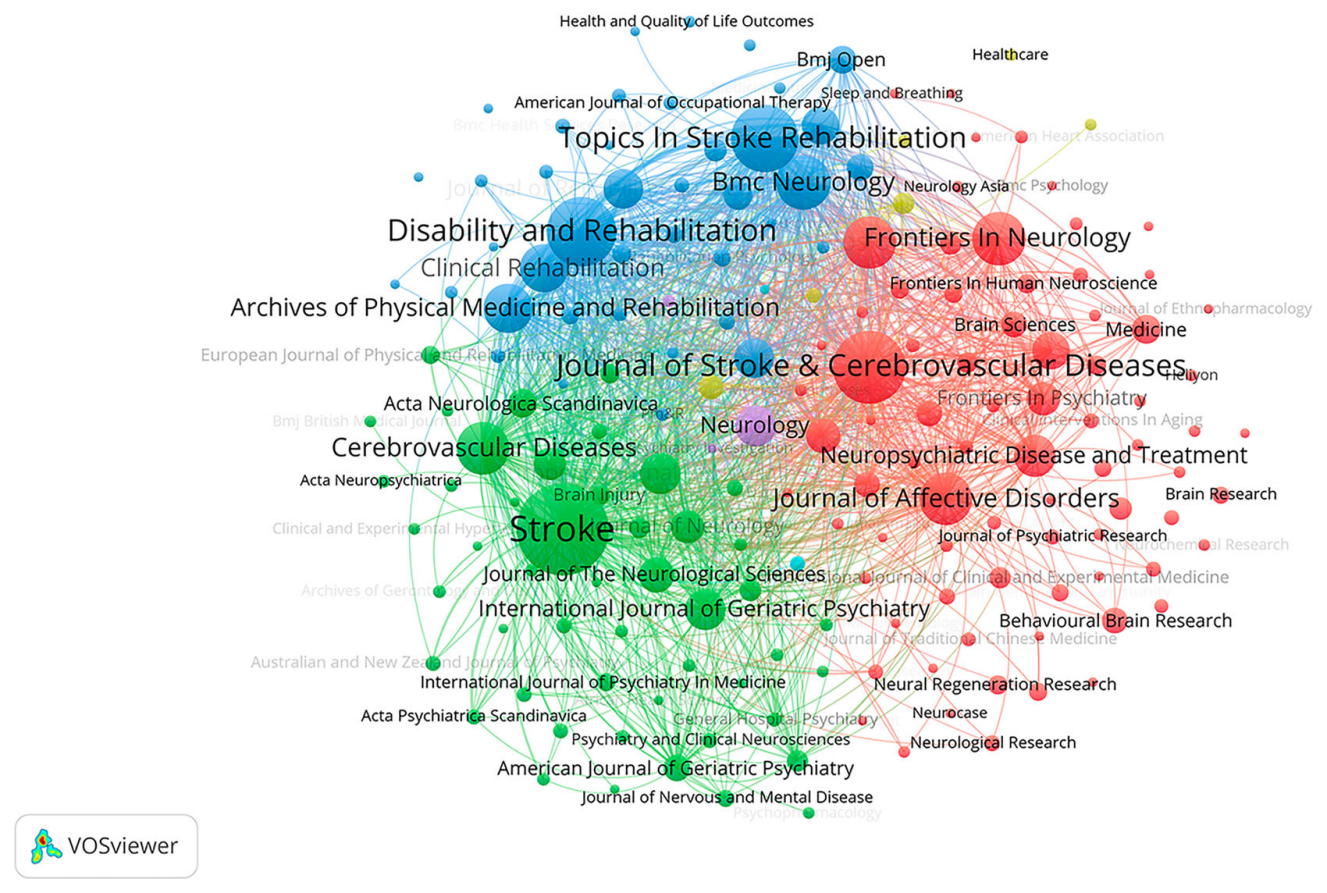
Clustered co‐occurrence of research journals.

Figure 6, the stacked view of annual journal publication volume, Figure 7, the journal co‐occurrence clustering map, Figure 7, the journal co‐occurrence temporal view, and Table 3 collectively reveal the overall publication trend, static collaborative structures, core journal contributions, and dynamic temporal evolution of PSD research over the 40‐year study period. These resources synergistically illustrate the hierarchical network of journals and their evolving roles in disseminating PSD‐related research.

**TABLE 3:** Top 15 high‐output journals.

Ranking	Source	Documents	Citations
1	*Stroke*	152	16,228
2	*Journal of Stroke & Cerebrovascular Diseases*	103	1493
3	*Disability and Rehabilitation*	96	3637
4	*Topics in Stroke Rehabilitation*	92	1736
5	*BMC Neurology*	65	1337
6	*Frontiers in Neurology*	63	596
7	*Cerebrovascular Diseases*	61	3588
8	*Journal of Affective Disorders*	60	1492
9	*PLOS One*	60	1634
10	*Archives of Physical Medicine and Rehabilitation*	54	3043
11	*Clinical Rehabilitation*	53	2009
12	*Neuropsychiatric Disease and Treatment*	42	414
13	*European Journal of Neurology*	39	1602
14	*International Journal of Geriatric Psychiatry*	39	1276
15	*Neurology*	39	2083

Figure 7 employs VOSviewer‐based hotspot clustering to visualize the thematic clustering structure and collaborative interconnections among journals. Three distinct core clusters characterize the global journal collaboration landscape. The first and largest cluster centers on *Stroke*, which contributes 152 publications as detailed in Table 3. *Stroke* serves as the core node of this cluster with dense collaborative links to numerous specialized journals, reflecting its dominant position in integrating cerebrovascular disorder research, including PSD. The second cluster is led by the *Journal of Stroke & Cerebrovascular Diseases* with 103 publications, as documented in Table 3. This cluster includes journals focused on stroke‐related complications and features strong inter‐journal cohesion. The third cluster revolves around *Disability and Rehabilitation* with 96 publications and *Topics in Stroke Rehabilitation* with 92 publications, as indicated in Table 3. This cluster focuses on rehabilitation‐oriented research and exhibits close collaborative connections within the rehabilitation research community. *BMC Neurology* with 65 publications, *Frontiers in Neurology* with 63 publications, and *Cerebrovascular Diseases* with 61 publications form secondary nodes across the three clusters, enhancing the overall connectivity of the global journal network.

Figure 6, the stacked view of annual journal publication volume quantifies the temporal variation of total PSD‐related publications and the contribution of each journal cluster to annual output. The stacked view clearly shows a steady growth in annual publication volume from 1985 to 2000, followed by a rapid surge after 2010. It further reveals the changing proportion of publications from different journal clusters over time. The cluster led by *Stroke* maintains the largest share of annual publications across most of the study period, consistent with its long‐standing core status. The rehabilitation‐focused cluster shows a gradual increase in its proportion of annual output from the mid‐2000s onwards, reflecting the growing academic attention to rehabilitation aspects of PSD. The cluster led by the *Journal of Stroke & Cerebrovascular Diseases* exhibits a notable rise in contribution after 2015, aligning with the expanding focus on stroke complications.

Figure 7 the journal co‐occurrence temporal view complements Figures 6 and 7 by presenting the temporal distribution of individual journal activities through color gradients. Stroke maintains consistent high activity across the entire 1985–2024 period, demonstrating its long‐standing role as a cornerstone in the field—a pattern that is quantitatively supported by its sustained high contribution in Figure 6. *Journal of Stroke & Cerebrovascular Diseases* exhibits increased activity from the early 2000s onwards, with a gradual deepening of color in the temporal view that aligns with its growing publication output and rising proportion in the stacked view. *Disability and Rehabilitation* and *Topics in Stroke Rehabilitation* show sustained activity from the mid‐2000s, their growing share in Figure 6 correlating with the intensified color gradient in Figure 7. *BMC Neurology* and *Frontiers in Neurology* display a notable surge in activity from 2015 onwards, which corresponds to their expanding contribution in the stacked view and reflects the broader integration of neurological research with PSD themes.

### 3.5 Key Author Contributions

**FIGURE 8:**
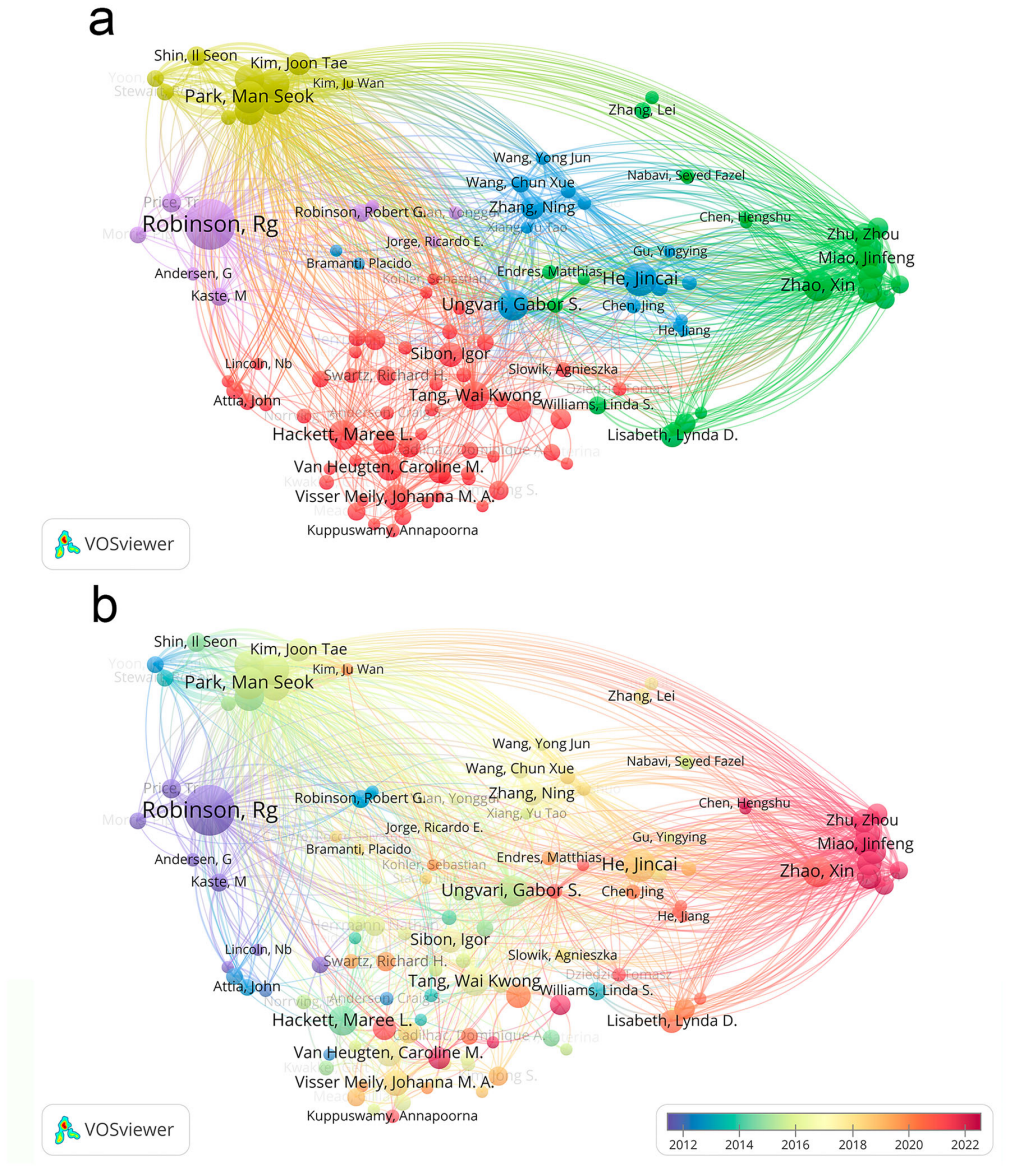
Study author collaboration information, (a) clustered co‐occurrence, (b) temporal co‐occurrence.

Figure 8a is the author co‐occurrence clustering diagram, Figure 8b is the author co‐occurrence time view, and Table 4 is the detailed information of the authors of the high publications. These collectively reveal the static collaborative clustering pattern, core author contributions, and dynamic temporal evolution of PSD research over a 40‐year study period. These VOSviewer‐based visualizations work synergistically to illustrate the hierarchical network of key authors and their evolving roles in shaping the field.

**TABLE 4:** Top 10 high‐output authors.

Ranking	Authors	Documents	Citations
1	R. G. Robinson	38	4184
2	Jincai He	24	428
3	Man Seok Park	23	715
4	Jae Min Kim	22	684
5	Gabor S. Ungvari	22	709
6	Xin Zhao	22	393
7	Ki Hyun Cho	21	706
8	Maree L. Hackett	21	1690
9	Hee Ju Kang	21	634
10	Sung Wan Kim	21	679

Figure 8a employs hotspot clustering to visualize the thematic clustering structure and collaborative interconnections among core authors. Four distinct core clusters characterize the global author collaboration landscape. The first and most influential cluster centers on R. G. Robinson, who contributed 38 publications. R. G. Robinson serves as the central node of this cluster with dense collaborative links to numerous researchers, reflecting his dominant position in integrating psychological and clinical aspects of PSD research. The second cluster is led by Jincai He with 24 publications, featuring strong internal cohesion and connections to researchers focused on epidemiological studies of PSD. The third cluster groups Man Seok Park with 23 publications and Jae Min Kim with 22 publications, forming a cohesive network centered on neurobiological mechanisms of PSD. The fourth cluster includes Gabor S. Ungvari with 22 publications, Xin Zhao with 22 publications, Ki Hyun Cho with 21 publications, and Maree L. Hackett with 21 publications. This cluster exhibits extensive cross‐cluster collaborative connections, highlighting the international collaborative nature of PSD research.

Figure 8b complements Figure 8a by presenting the temporal distribution of author research activities through color gradients. R. G. Robinson maintains consistent high activity across the entire 1985–2024 period, demonstrating his long‐standing and sustained contribution to the field. Jincai He shows a notable increase in research activity from 2010 onwards, with a distinct deepening of color in the temporal view that aligns with the expansion of PSD epidemiological research in China. Man Seok Park and Jae Min Kim exhibit stable research engagement from the mid‐2000s, their temporal color gradients reflecting continuous focus on neurobiological themes. Gabor S. Ungvari, Xin Zhao, and Ki Hyun Cho display sustained activity from the early 2010s, while Maree L. Hackett maintains steady contributions from the mid‐2010s onwards, reflecting the growing international collaboration in patient‐centred care and rehabilitation‐focused PSD research.

### 3.6 Keyword Analysis

**FIGURE 9:**
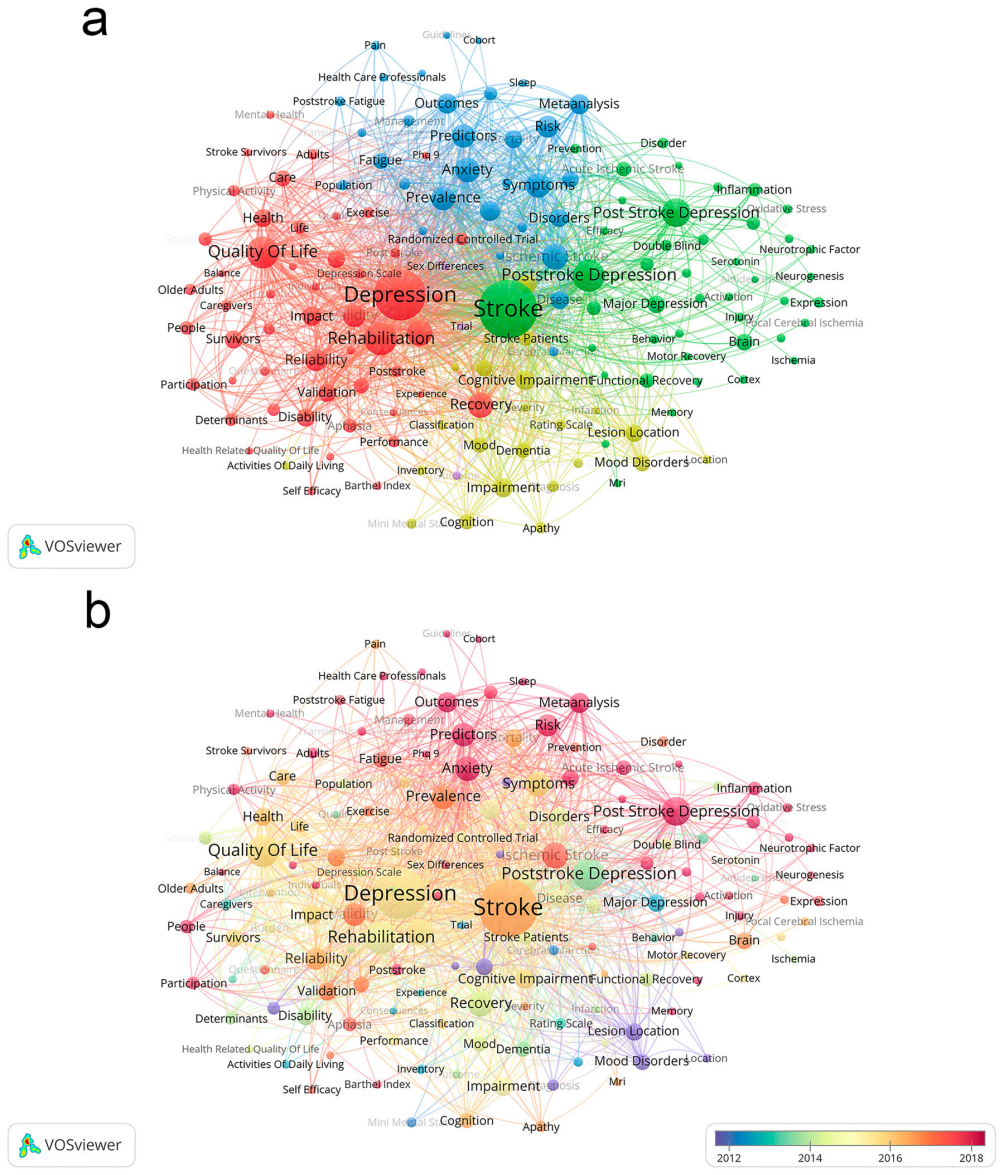
Keyword co‐occurrence network, (a) clustered co‐occurrence, (b) temporal co‐occurrence.

Figure 9a, the keyword co‐occurrence clustering map and Figure 9b, the keyword co‐occurrence temporal view, collectively reveal the static thematic clustering structure and dynamic temporal evolution of research foci in PSD studies over the 40‐year period. These VOSviewer‐based visualizations work synergistically to decode the knowledge framework of PSD research, with Figure 9a providing a snapshot of keyword collaborative networks and Figure 9b tracking the shifting intensity of research themes across time.

Figure 9a employs hotspot clustering to visualize the hierarchical thematic structure and interconnections among core keywords. Five distinct and cohesive clusters characterize the global PSD research landscape. The first and most central cluster is anchored by PSD (548) and depression (735), with strong connections to quality of life (545). This cluster forms the foundational conceptual framework of the field, linking the core condition to a key patient‐centered outcome. The second cluster centers on rehabilitation (311) and recovery (300), reflecting the integral role of rehabilitative interventions in PSD management. The third cluster groups predictors (275), risk factors (209) and prevalence (270), establishing the epidemiological backbone of PSD research. The fourth cluster includes cognitive impairment (192) and anxiety (159), highlighting the focus on comorbid conditions that modulate PSD outcomes. The fifth cluster revolves around scale (350), validity (246), and reliability (230), supplemented by meta‐analysis (210) and follow‐up (118), underscoring the emphasis on methodological rigor and evidence synthesis in the field.

Figure 9b complements Figure 9a by illustrating the temporal distribution of each keyword cluster through color gradients. The foundational cluster anchored by PSD and depression maintains consistent high activity across the entire 1985–2024 period, confirming its enduring centrality. The rehabilitation and recovery cluster exhibits a gradual increase in activity from the mid‐1990s onwards, with a notable intensification after 2010. The epidemiological cluster featuring predictors, risk factors, and prevalence rose to prominence in the early 2000s and remains highly active thereafter. The comorbidity‐focused cluster, including cognitive impairment and anxiety, gains traction from the late 2000s, reflecting growing recognition of multi‐morbidity in stroke populations. The methodological cluster centered on scale, validity, and reliability shows a marked surge in activity from 2015 onwards, aligning with the field's increasing emphasis on standardized assessment and evidence‐based practice.

### 3.7 Research Cluster Analysis

**FIGURE 10:**
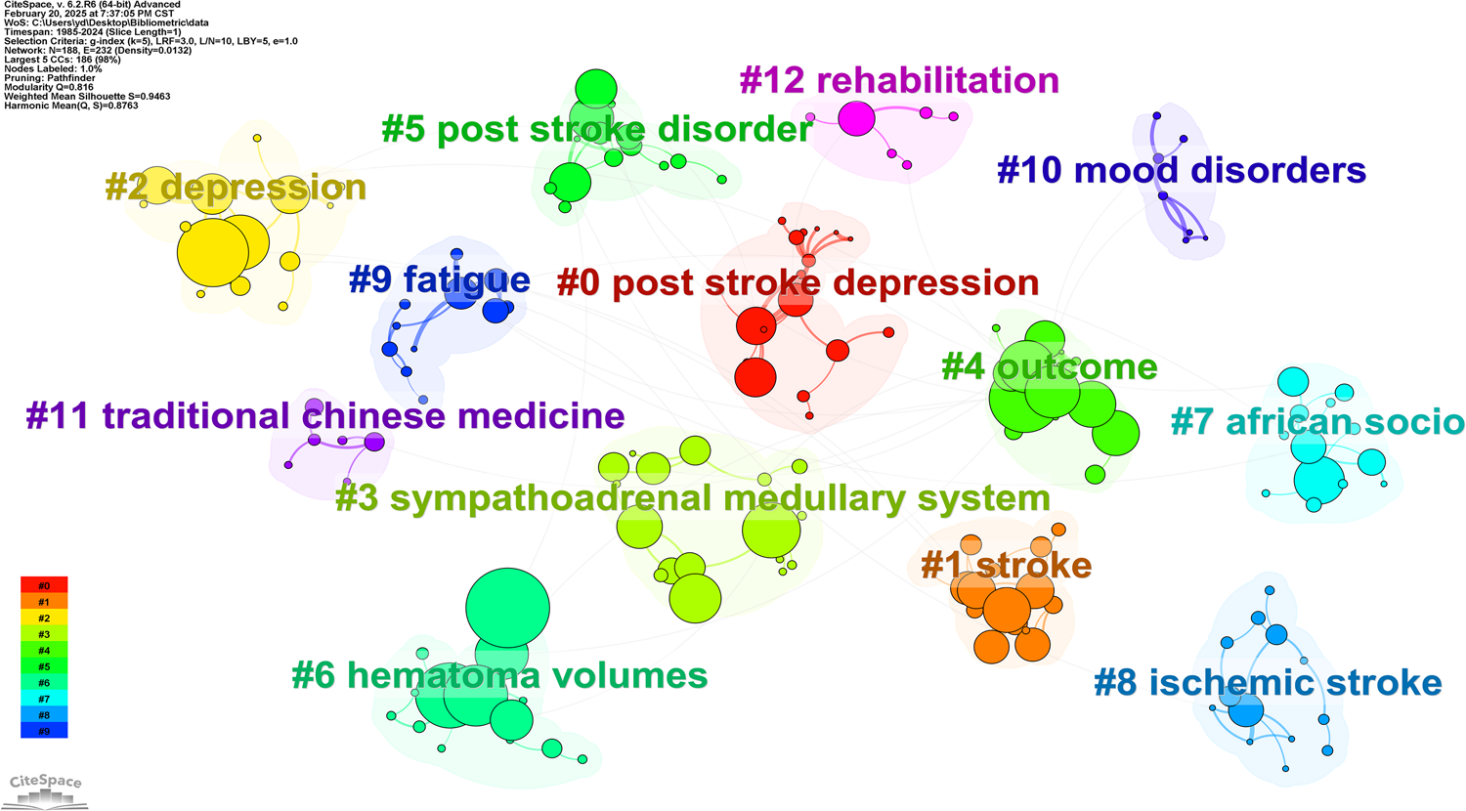
Cluster diagram of thematic research.

Figure 10 and Table 5 show that the bibliometric analysis of PSD from 1985 to 2024 reveals distinct clusters of research reflecting the evolving landscape of this critical area of stroke recovery and mental health.

**TABLE 5:** Keyword clustering parameters.

Cluster ID	Node number	Contour value	Year	Main keywords
0	20	0.986	2004	post‐stroke depression; vascular cognitive impairment; geriatric depression scale; regression analysis; prospective longitudinal study | depression; quality of life; disability; health; survivors
1	19	0.963	2011	stroke; quality of life; low‐income population; speech therapy; stroke epidemiology | post‐stroke depression; low‐income population; speech therapy; stroke epidemiology; psychometric properties
2	18	1	2003	stroke; depression; caregivers; spouse participation; balance impairment rehabilitation | quality of life; rehabilitation; impact; scale; physical activity
3	17	0.935	2008	post‐stroke depression; sympathoadrenal medullary system; vestibular disorders; Chinese herbal medicine; psychometric properties | ischemic stroke; neurofilament light; axonal injury; sympathoadrenal medullary system; vestibular disorders
4	17	0.954	2000	stroke; outcome; mood; prognostic; epidemiological | post‐stroke depression; acute phase; stroke unit; daily life activities; speech therapy
5	15	0.924	2008	post‐stroke depression; post stroke disorder; family functioning; nutrition surveys; post stroke epilepsy | risk factors; post stroke disorder; family functioning; nutrition surveys; post stroke epilepsy
6	15	0.937	2007	post‐stroke depression; post stroke disorder; hematoma volumes; Xingnao Jieyu capsule; Chinese herbal medicine | stroke; outcome; upper limb; self report; social competence
7	13	1	2011	post‐stroke depression; African socio; cultural context; vulnerable patients; modifiable characteristics | stroke; depression; anxiety; neuropsychology; disability
8	13	1	2011	post‐stroke depression; ischemic stroke; Jiedu Tongluo granules; traditional Chinese medicine; endocrine system | brain; neurotrophic factor; ptp1b; extracellular atp; microglial activation
9	12	0.986	2008	stroke; fatigue; depression; anxiety; cognition | follow‐up; rehabilitation; poststroke fatigue; validation; long
10	7	0.966	1992	mood disorders; location; follow‐up; prevalence; lesion | lesions; recovery; symptoms; post‐stroke depression; temporal lobe
11	6	0.975	2008	post‐stroke depression; traditional Chinese medicine; Jiedu Tongluo granules; magnetic resonance imaging; stroke rehabilitation | trial; disorders; risk factors; lesion location; post‐stroke depression
12	6	0.919	2017	stroke; rehabilitation; Africa; assessment; eye movement disorders | hospital anxiety; depression scale; risk factors; prevalence; cognitive impairment

Cluster 0 contains 20 keywords and has a silhouette score of 0.986, which indicates strong cohesion among the keywords. Identified in 2004, the cluster includes terms such as “post‐stroke depression,” “vascular cognitive impairment,” “Geriatric Depression Scale,” “regression analysis,” and “prospective longitudinal study.” The presence of these keywords suggests that this cluster primarily investigates the relationship between PSD and the cognitive impairments that often accompany stroke. The focus on the “Geriatric Depression Scale” highlights the importance of assessing depression in older adults, who are particularly vulnerable to both stroke and its psychological consequences. The association between “vascular cognitive impairment” and “post‐stroke depression” emphasizes the bidirectional relationship between cognitive decline and mood disorders, highlighting the need for integrated assessments in clinical settings.

*Cluster 1* contains 19 keywords and has a silhouette score of 0.963, emerged in 2011. Keywords include “stroke,” “quality of life,” “low‐income population,” “speech therapy,” and “stroke epidemiology.” The inclusion of “quality of life” highlights the increasing recognition of the importance of mental health in the overall well‐being of stroke survivors, especially those in economically disadvantaged populations. The keywords suggest that the focus of this research is on the challenges faced by low‐income stroke survivors, including limited access to rehabilitation services such as speech therapy, which can have a significant impact on recovery outcomes. The epidemiological perspective presented in this cluster further emphasizes the need for public health interventions that are tailored to vulnerable populations.

*Cluster 2* comprises 18 keywords and achieved a silhouette score of 1.0. It was identified in 2003. Key terms include “stroke,” “depression,” “caregivers,” “spouse participation,” and “balance impairment rehabilitation.” This cluster emphasizes the role of caregivers in managing PSD and highlights the emotional and psychological burden they face when supporting stroke survivors. The inclusion of the term “spouse participation” highlights the significance of family dynamics in the rehabilitation process, suggesting that supportive relationships can mitigate the impact of depression. Furthermore, the focus on “balance impairment rehabilitation” illustrates the connection between physical rehabilitation efforts and mental health outcomes, showing the interplay between physical recovery and psychological well‐being.

*Cluster 3*, with 17 keywords and a silhouette score of 0.935, this cluster emerged in 2008. It includes terms such as “post‐stroke depression,” “sympathoadrenal medullary system,” “vestibular disorders,” and “Chinese herbal medicine.” The inclusion of “sympathoadrenal medullary system” suggests a physiological focus on how stress responses may contribute to the development of depression following a stroke. The inclusion of “Chinese herbal medicine” indicates an interest in alternative therapies for managing PSD and reflects a growing trend towards integrative approaches in mental healthcare. The association between vestibular disorders and depression underscores the multifaceted nature of stroke recovery, as balance and spatial orientation issues can intensify feelings of helplessness and anxiety.

*Cluster 4* consists of 17 keywords and has a silhouette score of 0.954. It was identified in 2000. Keywords include “stroke,” “outcome,” “mood,” “prognostic,” and “epidemiological.” The cluster focuses on prognostic factors influencing mood disorders, particularly PSD, among stroke survivors. The emphasis on “outcome” indicates a keen interest in understanding the impact of mood on recovery trajectories and overall health outcomes. The epidemiological aspects emphasize the need for population‐level studies to identify trends and risk factors associated with PSD.

*Cluster 5* contains 15 keywords, has a silhouette score of 0.924, and emerged in 2008. It includes terms such as “post‐stroke depression,” “post‐stroke disorder,” “family functioning,” and “nutrition surveys.” This cluster emphasizes the importance of family dynamics and nutritional factors in the context of PSD. The focus on “family functioning” suggests that the quality of family relationships could be crucial for the psychological well‐being of stroke survivors. Furthermore, the inclusion of “nutrition surveys” suggests an interest in the potential influence of dietary factors on mental health.

*Cluster 6* was identified in 2007, with 15 keywords and a silhouette score of 0.937. It includes terms such as “post‐stroke depression,” “post‐stroke disorder,” “haematoma volumes,” and “Xingnao Jieyu capsule.” The focus on “haematoma volumes” suggests a physiological exploration of how stroke severity impacts the likelihood of developing depression. The inclusion of “Xingnao Jieyu capsule,” a traditional Chinese medicine, suggests an interest in alternative therapies for managing PSD.

*Cluster 7* contains 13 keywords, achieved a silhouette score of 1.0, and emerged in 2011. Key terms include “post‐stroke depression,” “African socio‐cultural context,” “vulnerable patients,” and “modifiable characteristics.” The cluster emphasizes the importance of cultural factors in understanding and addressing PSD, particularly within African populations. The focus on “vulnerable patients” emphasizes the need for culturally sensitive treatment and rehabilitation approaches.

*Cluster 8* comprises 13 keywords and has a silhouette score of 1.0. It emerged in 2011. It includes terms such as “post‐stroke depression,” “ischaemic stroke,” “Jiedu Tongluo granules,” and “traditional Chinese medicine.” The cluster focuses on applying traditional Chinese medicine to manage PSD in stroke survivors. The inclusion of “ischaemic stroke” suggests a particular interest in this type of stroke, which may be associated with higher rates of depression.

*Cluster 9* comprises 12 keywords and has a silhouette score of 0.986. It was identified in 2008. Key terms include “stroke,” “fatigue,” “depression,” “anxiety,” and “cognition.” The cluster illustrates the interconnectedness of fatigue, depression, and cognitive function in stroke survivors. The focus on “follow‐up” and “rehabilitation” highlights the commitment to understanding how these factors evolve, as well as the importance of providing patients with ongoing support.

*Cluster 10* emerged in 1992 with seven keywords and a silhouette score of 0.966. It includes terms such as “mood disorders,” “location,” “follow‐up,” and “prevalence.” The cluster suggests a focus on geographical and locational aspects of mood disorders, particularly in relation to stroke outcomes. The emphasis on “prevalence” indicates a desire to understand how mood disorders, including PSD, manifest in different populations and settings.

*Cluster 11* contains six keywords and has a silhouette score of 0.975. It was identified in 2008. It features terms such as “post‐stroke depression,” “traditional Chinese medicine,” “Jiedu Tongluo granules,” and “magnetic resonance imaging.” This cluster emphasizes the use of traditional remedies alongside advanced imaging techniques to improve understanding of PSD. Integrating these approaches reflects a multidisciplinary perspective that is vital for comprehensive care.

*Cluster 12* emerged in 2017 with six keywords and a silhouette score of 0.919. It includes terms such as “stroke,” “rehabilitation,” “Africa,” “assessment,” and “eye movement disorders.” The cluster emphasizes the importance of rehabilitation practices in Africa, particularly with regard to specific assessment tools for stroke‐related complications. The focus on “eye movement disorders” indicates a growing interest in the effects of stroke on sensory and perceptual functions, with further implications for mental health outcomes.

Examining the rich array of keywords across the clusters makes it clear that the study of PSD involves more than just addressing the psychological ramifications of a stroke; it also considers the myriad factors that influence recovery. These factors include the roles of caregivers, the influence of socioeconomic factors, the implications of various treatment modalities, and the necessity for culturally sensitive interventions. As research continues to advance, it is essential to prioritize comprehensive care models that address both the physical and mental health aspects of stroke recovery, thereby improving the quality of life for survivors.

### 3.8 Annual Development Analysis of Keywords

**FIGURE 11:**
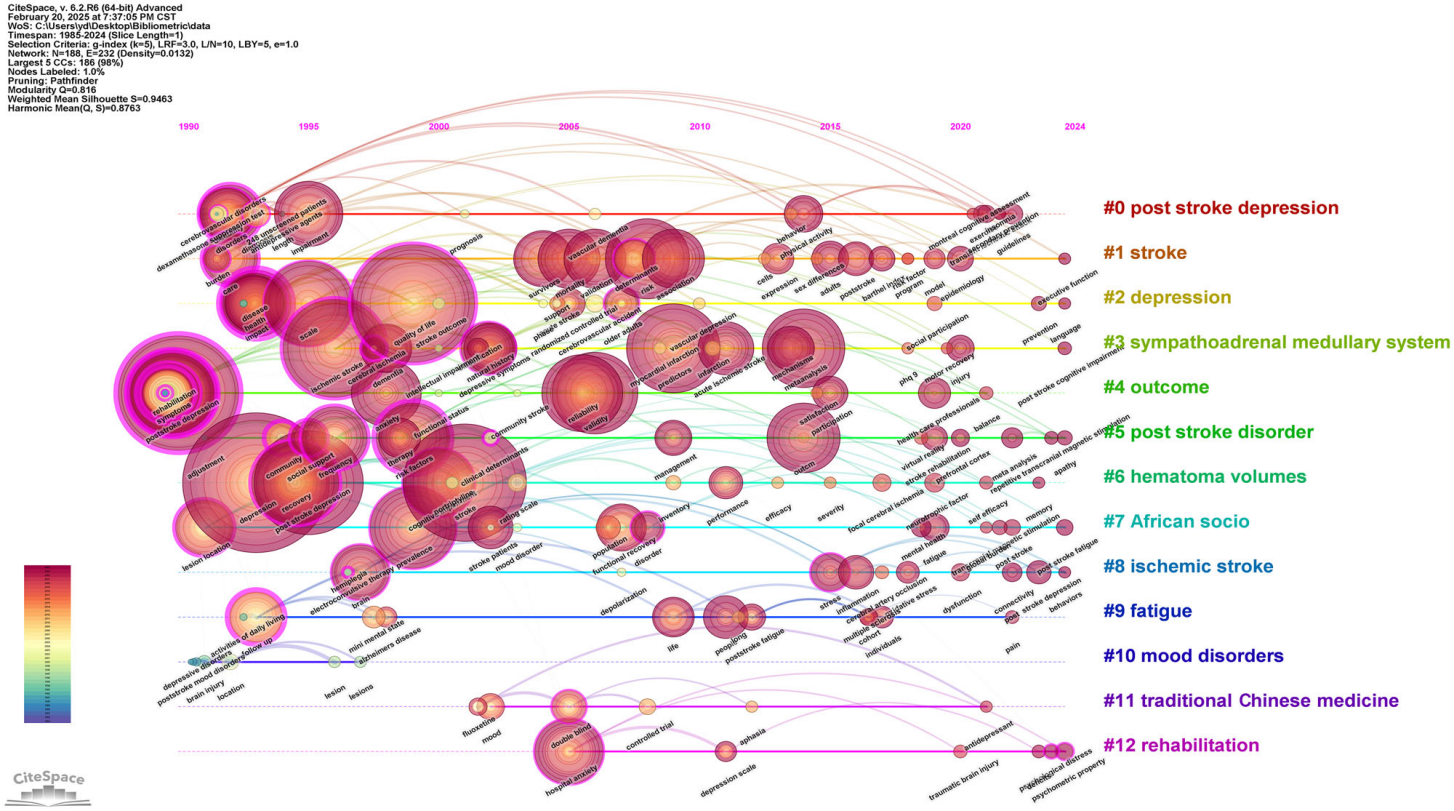
Timeline chart of the thematic study.

In the context of bibliometric analysis of PSD from 1985 to 2024, keyword analysis conducted through CiteSpace provided profound insights into trends and developments over the years, as shown in Figure 11 and Table 6.

**TABLE 6:** Keyword frequency and year.

Ranking	Keywords	Frequency	Year
1	Depression	735	1993
2	Post‐stroke depression	548	1990
3	Quality of life	545	1999
4	Stroke	505	2001
5	Post‐stroke depression	421	1995
6	Scale	350	1995
7	Ischemic stroke	346	1996
8	Symptoms	316	1990
9	Rehabilitation	311	1990
10	Recovery	300	1995
11	Predictors	275	2009
12	Prevalence	270	1999
13	Validity	246	2006
14	Risk	234	2008
15	Reliability	230	2006
16	Meta‐analysis	210	2014
17	Risk factors	209	1999
18	Impact	192	1993
19	Cognitive impairment	192	2000
20	Impairment	176	1995

Firstly, between 1990 and 1999, keywords such as “post‐stroke depression” (548), “quality of life” (545), and “rehabilitation” (311) became particularly prevalent. The early 1990s saw significant recognition of “post‐stroke depression,” highlighting the psychological challenges faced by stroke survivors. By 1999, the concept of “quality of life” had become increasingly relevant, reflecting a shift towards understanding the overall well‐being of stroke patients as more than just survival. During this period, “rehabilitation” was also established as a critical component of recovery, emphasizing the need for comprehensive care strategies that address both physical and mental health.

From 2000 to 2009, keywords such as “predictors” (275), “prevalence” (270), and “risk factors” (209) gained prominence. This period marked a shift towards identifying the predictors and prevalence of PSD, with researchers focusing more on the contributing factors. The emergence of “risk factors” as a key term indicated a growing interest in understanding the demographic and clinical characteristics that predispose individuals to depression following a stroke. During this period, “cognitive impairment” (192) also emerged as a relevant keyword, highlighting the link between cognitive challenges and the psychological outcomes experienced by stroke survivors.

Finally, from 2010 to 2024, keywords such as “meta‐analysis” (210), “validity” (246), and “reliability” (230) came to the fore. The 2010s saw significant advancements in research methodologies, with meta‐analyses highlighting the synthesis of findings across studies to draw robust conclusions about PSD. The focus on “validity” and “reliability” highlights the importance of creating reliable assessment tools for diagnosing and measuring depression in stroke patients. Furthermore, the emergence of keywords such as “hospital anxiety” (143) and “fatigue” (32) indicates a broader understanding of the comorbid conditions that often accompany PSD. This period reflects the maturation of research in this field, with an emphasis on evidence‐based practices and integrating mental health assessments into stroke rehabilitation programs.

### 3.9 Keywords With the Strongest Citation Bursts

**FIGURE 12:**
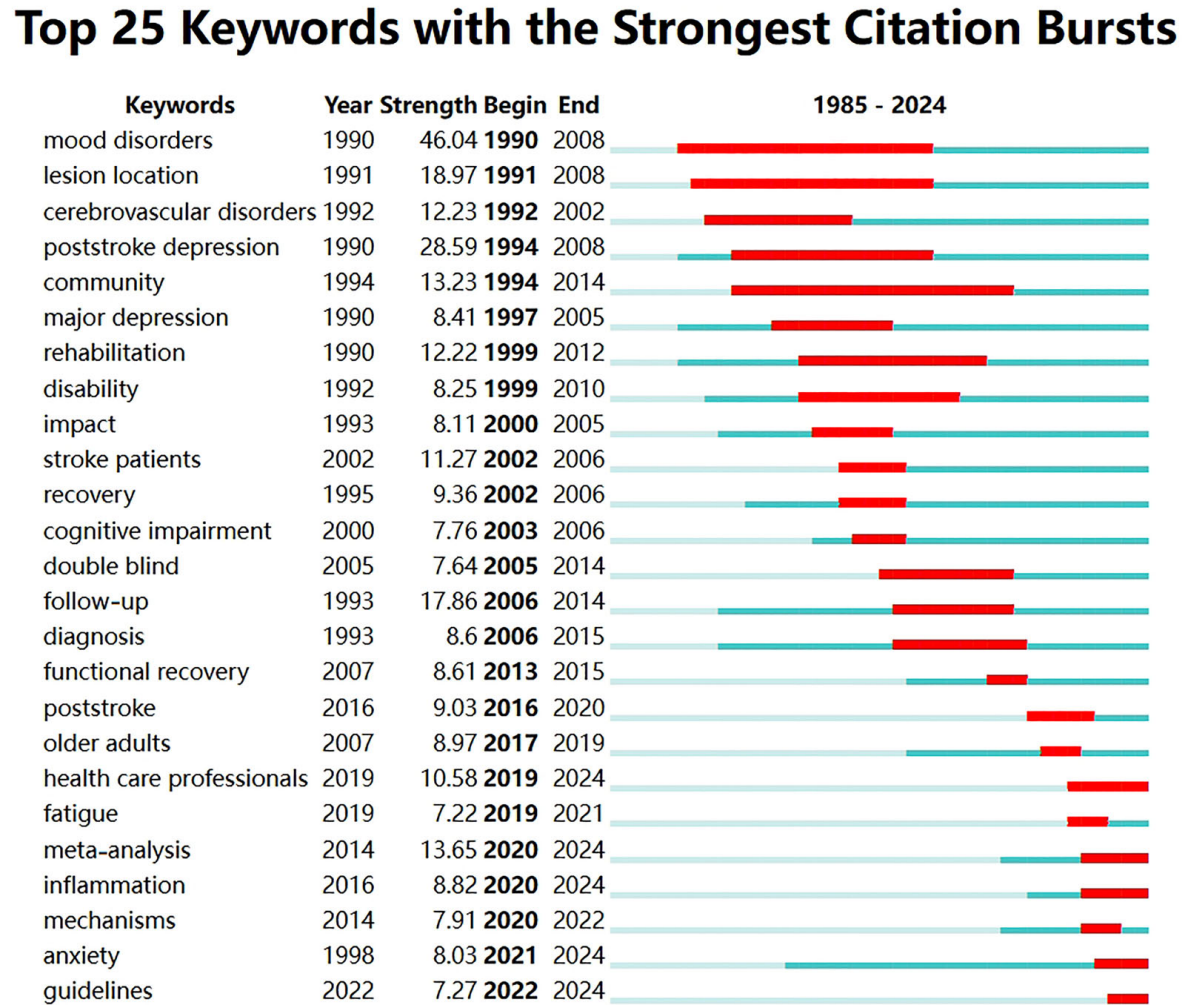
Keyword emergence analysis.

According to the results calculated by CiteSpace, the important emerging keywords in PSD research and their respective importance indices, start and end years are shown in Figure 12. First, the keyword “mood disorders,” which emerged in 1990 with a strength of 46.04, highlights the early recognition of the psychological impact of stroke on patients. Its early emergence highlights the initial recognition of mood disorders in stroke recovery and sets the stage for subsequent research on PSD. Its sustained relevance until 2008 demonstrates the continuous focus on the psychological impact of strokes on individuals and the importance of addressing mood disorders in rehabilitation protocols.

The emergence of “post‐stroke depression” in 1990, with a strength of 28.59, signifies a growing awareness of the specific mental health challenges faced by stroke survivors. Spanning from 1994 to 2008, this keyword reflects the growing recognition of PSD as a significant research area, linking mood disorders directly to stroke recovery. The keyword “rehabilitation,” which emerged in 1990 with a strength of 12.22 and lasted until 2012, further emphasizes the holistic approach required for stroke recovery, in which both physical and mental health must be considered. This interconnectedness reveals that the rehabilitation process must incorporate strategies to manage physical recovery and mental well‐being, particularly in relation to mood disturbances.

As we move into the 2000s, the emergence of additional keywords such as “cerebrovascular disorders” (1992) and “community” (1994) highlights the broader context of post‐stroke recovery. The former indicates a focus on the underlying medical conditions that lead to strokes, while the latter emphasizes the importance of community support in the recovery process. Furthermore, the emergence of “follow‐up” in 2006, with a strength of 17.86, indicates a critical shift towards understanding the long‐term management of stroke patients. This emphasizes the need for ongoing assessment and support during the rehabilitation phase. This trend is complemented by the recognition of “functional recovery” in 2007, which reflects the increasing focus on improving the quality of life for stroke survivors.

In recent years, keywords such as “health care professionals” (2019), “fatigue” (2019), and “meta‐analysis” (2014) have emerged in PSD research, reflecting its evolving landscape. The appearance of “health care professionals” highlights the crucial role these individuals play in recognizing and managing PSD, which is essential for comprehensive patient care. In addition, the rise of “fatigue” in 2019 highlights another common issue faced by stroke survivors, suggesting that physical and mental fatigue are significant concerns that need to be addressed in rehabilitation settings. The emergence of “meta‐analysis” in 2014, with a strength of 13.65, indicates a shift towards synthesizing existing research findings to establish clearer guidelines and evidence‐based practices in the management of PSD.

From the early identification of mood disorders and PSD to a growing emphasis on rehabilitation and community support, research into stroke recovery has progressively adapted to its complexities. The interplay between physical recovery, mental health, and community involvement is crucial, emphasizing the need for an integrated approach to care. Moving forward, it is essential that researchers continue to monitor these emerging keywords and trends, integrating new findings and practices to improve the understanding and management of PSD in clinical settings.

### 3.10 Analysis of Highly Cited Literature

**FIGURE 13:**
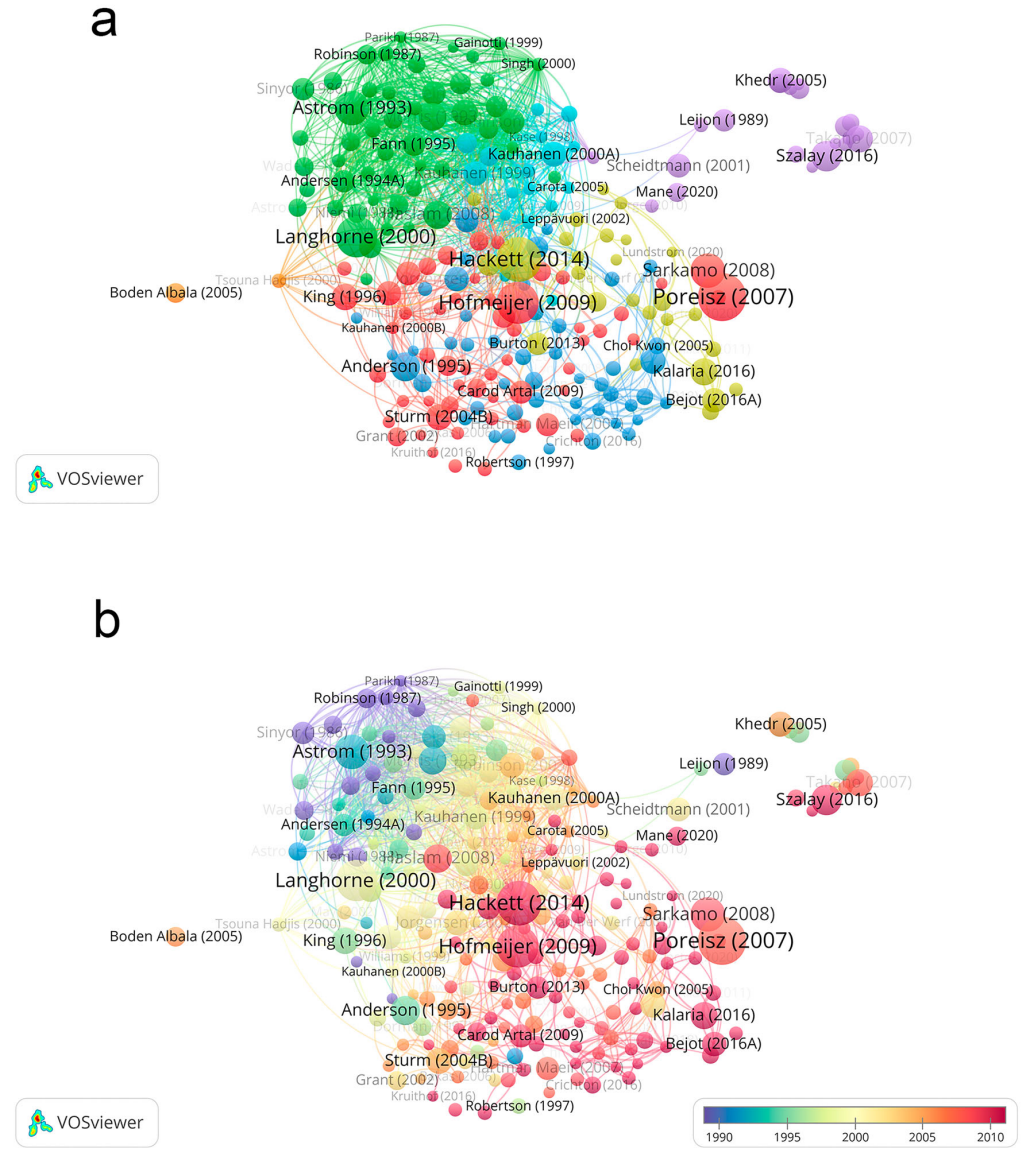
Highly cited local literature collaboration network. (a) Clustered co‐occurrence of local literature collaboration network, (b) temporal co‐occurrence of local literature collaboration network.

Figure 13 and Table 7 present the local citation results of bibliometric analyses on PSD published between 1985 and 2024, where Figure 13a,b serve complementary, non‐redundant roles that align closely with the textual interpretation of the literature landscape.

**TABLE 7:** Local literature is highly cited.

Ranking	Author (year)	Citations	DOI
1	Poreisz (2007)	810	https://doi.org/10.1016/j.brainresbull.2007.01.004
2	Hackett (2014)	742	https://doi.org/10.1111/ijs.12357
3	Hofmeijer (2009)	678	https://doi.org/10.1016/s1474 4422(09)70047 x
4	Langhorne (2000)	664	https://doi.org/10.1161/01.str.31.6.1223
5	Astrom (1993)	509	https://doi.org/10.1161/01.str.24.7.976
6	Sarkamo (2008)	505	https://doi.org/10.1093/brain/awn013
7	Szalay (2016)	432	https://doi.org/10.1038/ncomms11499
8	Anderson (1995)	402	https://doi.org/10.1161/01.str.26.5.843
9	Haslam (2008)	390	https://doi.org/10.1080/09602010701643449
10	Kalaria (2016)	362	https://doi.org/10.1016/j.bbadis.2016.01.015

Figure 13a is a VOSviewer‐based hotspot clustering map that identifies thematic clustering structures of core research elements and visualizes collaborative interconnections and cluster cohesion across the PSD field. This panel reveals distinct clusters of high‐impact studies: for example, the green cluster in the upper‐left quadrant centers on foundational epidemiological and clinical observations of PSD, anchored by Astrom (1993) (509 citations) and Langhorne (2000) (664 citations). The red‐orange cluster in the lower‐middle section aggregates research on therapeutic interventions and systematic reviews, with core nodes including Poreisz (2007) (810 citations), Hackett (2014) (742 citations), and Hofmeijer (2009) (678 citations). The yellow and purple clusters further delineate specialized themes, such as non‐pharmacological rehabilitation (e.g., Sarkamo [2008], 505 citations) and neurobiological mechanisms (e.g., Szalay [2016], 432 citations), respectively. These clusters collectively illustrate the modular yet interconnected structure of PSD research, with each group representing a sustained line of inquiry and collaborative network.

Figure 13b displays the VOSviewer‐based temporal distribution of research hotspots, using a color gradient (from blue to red) to reflect the publication years of core studies, thereby mapping the evolutionary trajectory of PSD research over time. The gradient reveals that early foundational work in the 1990s, such as Astrom (1993) and Anderson (1995) (402 citations), appears in cooler tones (blue‐green), marking the emergence of PSD as a distinct clinical concern. By the 2000s, warmer tones (yellow‐orange) highlight the expansion of research into therapeutic strategies and multicenter clinical investigations, exemplified by Langhorne (2000), Poreisz (2007), and Hofmeijer (2009). In the 2010s, the prevalence of red‐pink nodes signals a shift toward advanced neurobiological mechanisms (e.g., Szalay [2016]) and integrated care models (e.g., Kalaria [2016], 362 citations), demonstrating how research foci have evolved from initial epidemiological characterization to mechanistic exploration and holistic rehabilitation approaches.

The clustering patterns in Figure 13a and temporal trends in Figure 13b collectively contextualize the contributions of high‐impact studies. For instance, Astrom et al. (1993), a core node in the early green cluster, established the longitudinal prevalence of major depression post‐stroke, laying the groundwork for subsequent investigations into mental health screening in stroke populations. Building on this foundation, Langhorne (2000), located in the transitional yellow‐green cluster, expanded the scope to include medical complications and psychological distress, bridging physical and mental health outcomes in stroke recovery. The red cluster in Figure 13a, which includes Poreisz (2007) and Hackett (2014), reflects a mid‐2000s surge in intervention‐focused research: Poreisz (2007) provided critical safety data on transcranial direct current stimulation (tDCS) for neurorehabilitation, while Hackett (2014) synthesized prevalence data to inform clinical screening guidelines. Later studies, such as Sarkamo (2008) (music therapy) and Haslam (2008) (390 citations, social identity continuity), appear in the late‐2000s orange clusters, illustrating the diversification of non‐pharmacological and psychosocial approaches to PSD management. The 2010s pink nodes, including Szalay (2016) and Kalaria (2016), further demonstrate the field's progression toward understanding the neurobiological underpinnings of PSD and its links to long‐term cognitive outcomes, reinforcing the value of integrated, interdisciplinary care.

## 4 Discussion

### 4.1 Evolution of Research Focus and Thematic Dynamics

The 40‐year bibliometric trajectory of PSD research (1985–2024) reveals a clear evolutionary arc from fragmented observation to systematic, multidimensional inquiry—reflecting the field's growing recognition of PSD as a complex biopsychosocial disorder rather than a secondary complication of stroke. Early research (1985–1999) centered on foundational themes: the identification of PSD as a distinct clinical entity (evidenced by high‐frequency keywords like “post‐stroke depression” and “prevalence”), with landmark studies such as Astrom establishing its longitudinal incidence and clinical relevance. This phase aligned with broader advances in stroke care that shifted focus from mortality reduction to survivor well‐being, laying the groundwork for subsequent investigations into risk factors and outcomes.

By the 2000s, the field expanded to epidemiological characterization, with keywords like “predictors,” “risk factors,” and “cognitive impairment” emerging as core foci. This shift mirrored the accumulation of clinical data linking PSD to poorer functional recovery, highlighting the need to identify at‐risk populations—consistent with the clustering of research around comorbid conditions and demographic correlates. The 2010s onward marked a critical transition to intervention‐focused and methodologically rigorous research: the surge in “meta‐analysis,” “validity,” and “reliability” as high‐frequency terms reflects the field's push toward evidence‐based practice, while the diversification of clusters into non‐pharmacological therapies (e.g., music therapy, traditional Chinese medicine) and neurobiological mechanisms (e.g., microglial function, neurotransmitter imbalance) underscores a move beyond symptomatic management to targeted, mechanism‐driven care. This evolution aligns with global healthcare trends emphasizing holistic rehabilitation and personalized medicine, as seen in the AHA's 2020 guidelines, which integrate depression screening into mandatory stroke care.

### 4.2 Global Research Landscape and Collaborative Networks

The geographic and institutional distribution of PSD research highlights both strengths in global collaboration and persistent disparities. China and the United States, as the top two contributing countries (796 and 615 publications, respectively), dominate the research landscape—driven by their large aging populations, substantial investment in neurological research, and robust institutional networks. The University of Melbourne, Maastricht University, and King's College London emerge as core collaborative hubs, reflecting their long‐standing expertise in stroke rehabilitation and mental health integration. Notably, the clustering of country collaborations (Figure 4a) shows dense intra‐cluster connections within Chinese‐ and US‐led networks, with cross‐cluster links to European nations (e.g., England, Australia, the Netherlands)—indicating a degree of international knowledge exchange that has accelerated thematic progress, such as the adoption of standardized assessment tools across regions.

However, the underrepresentation of low‐ and middle‐income countries (e.g., Brazil, India) in top contributors reveals a critical gap: PSD disproportionately affects stroke survivors in resource‐limited settings, yet research from these regions remains scarce. This disparity is mirrored in thematic clusters, where “socioeconomic disparities” and “culturally sensitive interventions” are relatively underdeveloped compared to western‐focused clinical trials. The concentration of high‐impact journals (e.g., *Stroke*, *Journal of Stroke & Cerebrovascular Diseases*) in North America and Europe further reinforces this imbalance, as regional research may face barriers to dissemination in mainstream databases. Addressing this gap is not merely a matter of equity but clinical necessity: cultural factors, healthcare access, and comorbidity profiles (e.g., higher rates of untreated hypertension in low‐income regions) shape PSD presentation and outcomes, requiring context‐specific research to inform global care guidelines.

### 4.3 Translational Implications for Clinical Practice and Research Priorities

The bibliometric findings translate directly to actionable strategies for clinical practice, policy, and future research—rooted in the field's core strengths and identified gaps. For clinicians, the dominance of “rehabilitation,” “quality of life,” and “early screening” as key themes underscores the need to integrate mental health assessments into routine stroke care pathways. The high citation impact of studies like Hackett and Pickles, which synthesized PSD prevalence data to inform screening guidelines, validates the clinical utility of translating bibliometric insights into standardized protocols: routine use of tools like the Geriatric Depression Scale (a core keyword in Cluster 0) can improve early detection, reducing the risk of poor functional recovery and mortality associated with untreated PSD. In addition, the diversification of non‐pharmacological intervention research (e.g., music therapy, tDCS) provides clinicians with alternatives for patients with contraindications to SSRIs, addressing long‐standing debates about drug efficacy in PSD.

For researchers, emerging trends and thematic gaps point to clear priorities. Citation burst analysis identifies “healthcare professionals,” “fatigue,” and “meta‐analysis” as frontiers—signaling the need for studies that train stroke care teams in PSD management, explore comorbid symptoms (e.g., fatigue as a predictor of persistent depression), and synthesize existing evidence to resolve conflicting findings (e.g., the efficacy of pharmacological vs. non‐pharmacological interventions). The underdeveloped clusters around “family functioning,” “caregiver roles,” and “cultural context” also present opportunities: caregiver burden directly impacts patient recovery, yet research on supportive interventions for caregivers remains limited. Similarly, integrating traditional Chinese medicine (a recurring theme in Clusters 3, 8, and 11) with Western approaches requires rigorous multicenter trials to validate efficacy, addressing the current gap between regional practice and global evidence.

Notably, the temporal evolution of keywords (Figure 9b) shows a shift from descriptive epidemiology to mechanistic and translational research—indicating the field's maturation. Future studies should build on this trajectory by prioritizing interdisciplinary collaboration between neurologists, psychiatrists, rehabilitation specialists, and public health researchers. For policymakers, the concentration of research in high‐income countries highlights the need for targeted funding to support capacity building in low‐resource regions, ensuring that PSD research reflects the global burden of the condition. By aligning research priorities with clinical needs—rooted in the data‐driven insights of this bibliometric analysis—the field can advance toward more equitable, effective, and holistic care for stroke survivors with PSD.

## 5 Conclusion

A bibliometric analysis of research into PSD from 1985 to 2024 provides a comprehensive overview of evolving trends and key contributors in this important field of stroke recovery and mental health. The study reveals a significant increase in publication output over the past four decades, highlighting the growing recognition of PSD as a vital component of stroke rehabilitation. PSD research initially saw modest growth, with just five publications in 1985, but this figure steadily increased throughout the 1990s and early 2000s. A notable surge occurred in 1995 with 21 publications, and this upward trend continued, culminating in 295 publications in 2024.

China emerged as the leading country in PSD research, publishing 796 papers, followed by the United States with 615. These findings emphasize the substantial contributions of these countries, particularly China's extensive network of researchers and the United States' long‐standing leadership in medical research. Other notable contributors include England, Australia, the Netherlands, Germany, and Canada, which have each provided valuable insights through their research into the epidemiology, clinical characteristics, and treatment options for PSD. Further analysis reveals the pivotal role of key institutions such as the University of Melbourne, Maastricht University, and King's College London in advancing the understanding and management of PSD through multidisciplinary approaches and innovative research. Prominent authors, such as R. G. Robinson, Jincai He, and Man Seok Park, have had a significant influence on the field through their extensive publications focusing on the psychological implications of stroke, epidemiological studies, and the neurobiological links between stroke and depression.

Keyword analysis and clustering provide a detailed understanding of the research focus and thematic connections within PSD literature. Keywords such as “depression,” “post‐stroke depression,” “quality of life,” “rehabilitation,” and “recovery” emphasize the central themes of PSD research and highlight the need for comprehensive care models that address physical and psychological rehabilitation. Clustering analysis reveals distinct clusters of research, each focusing on a unique aspect of PSD. These include the relationship between PSD and cognitive impairments (Cluster 0), the impact of socioeconomic factors on mental health (Cluster 1), the role of caregivers and family dynamics (Cluster 2), and the exploration of alternative therapies, such as traditional Chinese medicine (Cluster 3). Together, these clusters emphasize the multifaceted nature of PSD and the importance of an integrative approach to managing this condition.

The keyword co‐occurrence network further illustrates the interconnectedness of these themes. It highlights how the focus of PSD research has evolved from initial recognition of PSD to identifying predictors and risk factors, and finally to emphasizing rigorous methodologies and comorbid conditions. Analyzing keywords with the strongest citation bursts reveals emerging trends and research frontiers. Recent years have shown a growing interest in topics such as “health care professionals,” “fatigue,” and “meta‐analysis.”

## 6 Relevance for Clinical Practice

This study underscores the importance of integrating mental health assessments into stroke care. It highlights early screening, personalized rehabilitation, and interdisciplinary approaches as key to improving PSD outcomes. Clinicians should address psychosocial factors and explore alternative therapies to enhance recovery. By adopting holistic rehabilitation models, healthcare providers can improve both the mental and physical well‐being of stroke survivors, ultimately leading to better long‐term recovery and quality of life.

## Author Contributions

All authors contributed to the study conception and design. **Xinjuan Wang**: writing – review and editing, writing – original draft, visualization, investigation. **Yinfen Qian**: writing – review and editing. **Qi He**: writing – review and editing, visualization, methodology, investigation, conceptualization. All authors read and approved the final manuscript.

## Funding

The authors have nothing to report.

## Ethics Statement

The authors have nothing to report.

## Consent

The authors have nothing to report.

## Conflicts of Interest

The authors declare no conflicts of interest.

## Data Availability

Data will be made available upon reasonable request.
